# Bidirectional Relationship Between Anaesthetic Drugs and Metabolic Syndrome: Clinical Effects and Mechanisms

**DOI:** 10.1111/jcmm.70905

**Published:** 2025-10-22

**Authors:** Yuying Huang, Qinghai Lan, Yijian Chen, Youchun Li, Baolin Zhong, Yuxin Zhan, Simin Deng

**Affiliations:** ^1^ Department of Anesthesiology Ganzhou People's Hospital Ganzhou Jiangxi People's Republic of China; ^2^ The Second Clinical Medical College, Jiangxi Medical College Nanchang University Nanchang China

**Keywords:** anaesthetic agents, insulin resistance, metabolic syndrome (MS)

## Abstract

Metabolic syndrome (MS) is a multifaceted condition characterised by insulin resistance, dyslipidemia, central obesity and hypertension, significantly elevating the risks of cardiovascular diseases and type 2 diabetes. This review aims to elucidate the bidirectional interplay between anaesthetic agents and MS, highlighting the mutual influence on metabolic regulation and anaesthetic efficacy. Accumulating evidence underscores the disruptive impact of anaesthetic agents on metabolic pathways. General anaesthetics can impair insulin signalling, thereby influencing insulin sensitivity, while local anaesthetics may indirectly affect systemic metabolism via alterations in local metabolic processes and blood flow. For instance, lidocaine interferes with the overall metabolism in the body by inhibiting tissue perfusion and local metabolic processes such as insulin signalling pathways. Conversely, MS can modulate the metabolism and efficacy of anaesthetic drugs, further complicating their clinical application. This review systematically explores the intricate relationship between anaesthetic agents and MS. It begins by delineating the primary features of MS and its potential impact on anaesthetic pharmacology. Subsequently, it examines the effects of diverse anaesthetic agents on components of MS, including insulin resistance, lipid metabolism and blood pressure regulation. Lastly, the review addresses how MS influences the metabolism and pharmacodynamics of anaesthetic drugs, offering insights into future research and clinical strategies to enhance anaesthetic management in MS patients.

**Abbreviations**
ER stress: endoplasmic reticulum stress
GIR: glucose infusion rate
MS: metabolic syndrome
OGTT: Oral Glucose Tolerance Test
PPIs: proton pump inhibitors
ROS: reactive oxygen species
TG: triglycerides

## 1 Introduction

Metabolic syndrome (MS) is a chronic, non‐infectious syndrome that aggregates a cluster of metabolic disorders, including insulin resistance, atherogenic dyslipidemia, central obesity and hypertension [1, 2]. This condition not only significantly elevates the risks of cardiovascular diseases, type 2 diabetes and other metabolic‐related disorders but also poses a formidable challenge to global public health [3]. Patients with metabolic syndrome exhibit complex imbalances in metabolic regulation [4], and studies indicate that metabolic syndrome is a significant factor contributing to surgical mortality [5], necessitating heightened attention to these patients' metabolic states in clinical practices and treatments, particularly in surgical and anaesthetic management.

Anaesthetic agents are indispensable tools in surgical and other invasive procedures. In recent years, mounting evidence suggests that these agents not only influence anaesthetic effects but may also disrupt metabolic processes [6]. For instance, certain general anaesthetic agents can interfere with insulin signalling pathways, altering insulin sensitivity [7], while local anaesthetics might indirectly impact systemic metabolism by affecting local metabolism and blood flow [8]. Additionally, metabolic syndrome itself can modify the metabolism and efficacy of anaesthetic drugs, complicating anaesthetic management [9].

In this context, this review aims to comprehensively investigate the bidirectional interactions between anaesthetic agents and metabolic syndrome. It seeks to elucidate the effects of anaesthetic drugs on metabolic syndrome and vice versa, thereby exploring the intricate relationship between the two. Initially, we will discuss the primary characteristics of metabolic syndrome and its potential impact on anaesthetic drugs. Subsequently, the review will delve into the effects of various types of anaesthetic agents on the components of metabolic syndrome, encompassing insulin resistance, body weight and lipid metabolism, and alterations in blood pressure.

Finally, we will explore how metabolic syndrome influences the metabolism and pharmacodynamics of anaesthetic drugs, providing future research directions and clinical application recommendations to optimise anaesthetic management in patients with metabolic syndrome.

## 2 Overview of Metabolic Syndrome

Metabolic syndrome (MetS) is an assemblage of interrelated conditions that frequently co‐occur, encompassing obesity, insulin resistance, impaired glucose tolerance, hypertension and dyslipidemia. This cluster is strongly associated with an increased incidence of diabetes and a heightened risk of cardiovascular events, such as heart disease and stroke, which have emerged as significant public health concerns [10].

Insulin resistance, a pivotal feature of MetS, is defined physiologically as the inability of certain tissues to respond effectively to normal insulin levels, necessitating elevated insulin levels to maintain metabolic functions [11]. In humans, this condition results in reduced glucose uptake by muscle, augmented hepatic glucose production (HGP) and increased lipolysis in adipose tissue [12]. A cascade of dysfunctional adipose tissue, lipotoxicity, impaired insulin signalling, glucotoxicity, oxidative stress and low‐grade inflammation further exacerbates the risk of diabetes [13]. Insulin resistance is multifactorial, involving obesity, dietary factors and chronic low‐grade inflammation with proinflammatory cytokine production.

Clinically, insulin resistance is assessed using techniques such as the Hyperinsulinemic‐Euglycemic Clamp (HEC) technique, the Oral Glucose Tolerance Test (OGTT) and the Homeostatic Model Assessment of Insulin Resistance (HOMA‐IR) [14].

Obesity, another critical marker of MetS, is intimately associated with insulin resistance [15] and cardiovascular disease through dysfunctional adipose tissue [16]. The World Health Organization (WHO) classifies overweight as a Body Mass Index (BMI) between 25 and 29.9 kg/m^2^, and obesity as a BMI ≥ 30 kg/m^2^, further subdivided into Class I (BMI = 30–34.9 kg/m^2^), Class II (35–39.9 kg/m^2^) and Class III (≥ 40 kg/m^2^) [17]. Assessment of fat distribution is often recommended using waist circumference (WC), with waist‐to‐height ratio (WHtR) and WC divided by height 0.5 (WHR.5R) proposed as improved estimators of relative abdominal fat [18].

Hypertension, a key component of MetS, is diagnosed with systolic blood pressure ≥ 130 mmHg and/or diastolic blood pressure ≥ 85 mmHg [19]. It is closely linked to other features of MetS, such as insulin resistance and abdominal obesity, significantly increasing the risk of cardiovascular events [20]. Effective management of hypertension is crucial for mitigating the overall risk associated with MetS.

Dyslipidemia, another significant feature of MetS, involves abnormal elevations in lipid levels, including triglycerides (TG) and low‐density lipoprotein cholesterol (LDL‐C), alongside decreased high‐density lipoprotein cholesterol (HDL‐C). This lipid abnormality is closely associated with the occurrence of cardiovascular diseases and is clinically assessed and managed through lipid testing [21].

In addition to these core features, MetS may also present with other symptoms, such as fatty liver, hyperuricemia and oxidative stress. Fatty liver is closely associated with MetS, potentially leading to liver dysfunction and increased diabetes risk [22]. Oxidative stress, a pathological foundation of MetS, promotes inflammatory responses and cellular damage, impacting metabolic health [23]. The relationship between hyperuricemia and MetS is also gaining attention, as elevated uric acid levels may negatively affect metabolic health [24].

In summary, MetS represents a complex pathological state involving multiple metabolic abnormalities. A deeper understanding of this syndrome is essential for devising more effective therapeutic and management strategies, thereby reducing associated disease risks and improving patient quality of life.

## 3 Impact of Anaesthetics on Insulin Resistance in Metabolic Syndrome

Insulin resistance, a hallmark of metabolic syndrome, plays a crucial role in glucose homeostasis. Anaesthetic agents may disrupt insulin function through various mechanisms, thereby affecting postoperative blood glucose levels.

### 3.1 Anaesthetics

#### 3.1.1 General Anaesthetics

##### 3.1.1.1 Isoflurane

Isoflurane is recognised for its detrimental effects on insulin secretion and glucose utilisation [25], impacting insulin resistance at multiple levels of peripheral and central molecular metabolism. Studies have shown that isoflurane exposure induces hyperglycemia, hyperinsulinemia and increases the Homeostatic Model Assessment of Insulin Resistance (HOMA‐IR) index, indicating increased peripheral insulin resistance [26]. Mechanically, isoflurane anaesthesia has been shown to induce hepatocyte necrosis and apoptosis by suppressing the expression levels of insulin‐like growth factor 1 (IGF‐1) and its receptor IGF‐R [27]. Furthermore, isoflurane impairs glucose tolerance by increasing hepatic glucose production and reducing peripheral glucose utilisation [28]. Insulin activates tyrosine kinase upon binding to the insulin receptor (IR), promoting the phosphorylation of insulin receptor substrate (IRS) proteins and the activation of downstream molecules such as phosphatidylinositol 3‐kinase (PI3K) and Akt (also known as protein kinase B) [29]. Isoflurane anaesthesia can inhibit the expression of PI3K and Akt [7], with Akt2 gene knockout mice exhibiting insulin resistance and type 2 diabetes phenotypes [30]. Missense mutations in the PKB (Akt2) kinase domain in humans are associated with severe insulin resistance, as these mutations prevent kinase phosphorylation of downstream targets and the inhibition of phosphoenolpyruvate carboxykinase (PEPCK), a key enzyme in gluconeogenesis [31]. In mice with pre‐existing insulin resistance induced by a high‐fat diet/streptozotocin, isoflurane anaesthesia significantly exacerbates central insulin resistance, particularly at the level of gene transcription and protein expression. Isoflurane exposure alters insulin signalling pathways in the frontal cortex and hippocampus of mice, with upregulation of phosphorylated insulin receptor substrate‐1 (pIRS1) and phosphorylated insulin receptor substrate‐2 (pIRS2), and downregulation of phosphorylated protein kinase B (pAKT) and phosphorylated glycogen synthase kinase‐3β (pGSK3β) [32]. Studies by Peng et al. further support the impact of isoflurane on central insulin resistance. Long‐term isoflurane exposure induces peripheral and central insulin resistance in adult mice and exacerbates insulin resistance in type 2 diabetes mice. Clinically, in a randomised single‐blind clinical trial, the anaesthesia protocol was similar in two groups except for the maintenance of anaesthesia (propofol in one group and isoflurane in the other). The results showed that 60 and 90 min after starting the operation, the blood glucose in the isoflurane group was significantly higher than that in the propofol group. This indicates that isoflurane can lead to an increase in blood glucose during anaesthesia for abdominal hysterectomy in diabetic patients [33].

##### 3.1.1.2 Sevoflurane

Sevoflurane anaesthesia severely impairs insulin‐stimulated whole‐body glucose uptake, as evidenced by a 50% reduction in glucose infusion rate (GIR), inducing insulin resistance in mice and reducing glucose uptake in insulin‐sensitive tissues such as the soleus, triceps brachii and gastrocnemius, while also reducing glucose uptake in non‐insulin‐sensitive tissues (e.g., brain) [34]. During sevoflurane anaesthesia, plasma free fatty acids (FFA) increased by 42% [32], and FFA, a potent inducer of insulin resistance, suggest that sevoflurane may affect insulin resistance by inducing FFA mobilisation [35]. Compared with other anaesthetics, the impact of sevoflurane on insulin resistance shows certain similarities and heterogeneities. Sevoflurane anaesthesia has a rapidly reversible inhibitory effect on basal and glucose‐stimulated insulin secretion, similar to other inhalational anaesthetics and may induce insulin resistance [36]. Sevoflurane anaesthesia, like isoflurane anaesthesia, has been shown to cause severe liver damage and insulin resistance [31, 37]. A study comparing the effects of sevoflurane and desflurane anaesthesia on blood glucose levels found that both sevoflurane and desflurane impaired glucose tolerance in a concentration‐independent manner [36]. Another randomised, single‐blind study confirmed that, compared to isoflurane, sevoflurane induces a lower incidence of postoperative hyperglycemia in non‐cardiac surgical patients (18% vs. 27%), but still significantly elevates blood glucose for up to 48 h in diabetic individuals. However, compared with desflurane, sevoflurane anaesthesia resulted in a more significant increase in blood glucose levels in non‐diabetic patients [38, 39, 40]. In summary, sevoflurane anaesthesia has a significant impact on insulin secretion and insulin resistance, potentially leading to increased insulin resistance and blood glucose levels. These findings are important for understanding the metabolic effects of sevoflurane in clinical anaesthesia.

##### 3.1.1.3 Propofol

Propofol anaesthesia induces whole‐body insulin resistance by significantly reducing the GIR, characterised by reduced insulin‐stimulated glucose uptake in skeletal muscle and myocardium, and weakened insulin‐mediated inhibition of hepatic glucose output [41, 42]. Besides its anaesthetic effects, propofol has been found to possess anti‐inflammatory and antioxidant properties, which can positively or negatively affect insulin resistance [43, 44, 45, 46]. On one hand, propofol significantly reduces the phosphorylation levels of Akt (Ser473) and GSK‐3β(Ser9) in cultured primary mouse hepatocytes, inhibiting the PI3K/Akt/GSK‐3β signalling pathway and thereby inhibiting hepatocyte glycogen synthesis, leading to insulin resistance in primary mouse hepatocytes [47]. Additionally, propofol treatment significantly increases the expression of protein and mRNA PTEN in primary mouse hepatocytes, and knocking out PTEN can reverse propofol‐induced insulin resistance in these cells [48]. On the other hand, propofol activates antioxidant enzymes such as superoxide dismutase (SOD) and glutathione peroxidase (GSH‐Px), reducing the production and accumulation of reactive oxygen species (ROS), thereby lowering oxidative stress levels and improving insulin signalling, alleviating insulin resistance [49]. The impact of different concentrations of propofol general anaesthesia on perioperative insulin resistance and inflammatory responses in patients undergoing elective abdominal surgery has been studied, with the high‐concentration group showing significantly lower levels of serum TNF‐α and IL‐6 postoperatively, while IL‐10 levels were significantly higher, indicating that propofol may affect insulin resistance by regulating the levels of inflammatory cytokines [47]. A clinical trial with prospective randomised control indicated that blood glucose levels showed a statistically significant increase at postoperative day 1 compared to the preoperative period after the propofol anaesthesia (150.2 ± 43.1 vs. 121.0 ± 25.9, *p* < 0.01) [50]. In summary, propofol, through its anti‐inflammatory and antioxidant actions, has a certain impact on insulin resistance, possibly by modulating inflammatory factors and insulin signalling pathways. These actions may help improve insulin resistance, especially during surgery and anaesthesia.

##### 3.1.1.4 Ketamine

Ketamine significantly inhibits insulin secretion [51] and increases insulin resistance in skeletal muscle cells [52], characterised by impaired insulin signalling and reduced glucose uptake, leading to significantly elevated blood glucose levels in normal or diabetic patients during anaesthesia induction and maintenance [53, 54]. Beta cells, as specialised secretory cells with well‐developed endoplasmic reticulum and various mechanisms to handle the folding demands of insulin proteins [55, 56], can induce endoplasmic reticulum stress (ER stress) under conditions of insulin resistance or β‐cell mass loss, leading to impaired insulin secretion [57]. Furthermore, ketamine‐induced mitochondrial dysfunction may affect the cellular environment by altering mitochondrial morphology (fusion or fission) [58]. Under stress, mitochondria fragment into smaller pieces, accelerating the production of intracellular ROS and reducing insulin secretion. In addition, ketamine can affect insulin resistance by regulating the levels of inflammatory factors [59, 60]. Specifically, ketamine inhibits inflammation mediated by pro‐inflammatory cytokines such as TNFα, IL1β and IFNγ, which are associated with M1 iPSC‐Mac‐induced iPSC‐Heps inflammation and insulin resistance [61]. In obesity‐induced inflammation, ketamine activates JNK and related pathways, leading to impaired glucose and lipid metabolism, blocking insulin signalling and reducing insulin sensitivity in target cells, a significant cause of type 2 diabetes (T2D) [62]. Ketamine is closely linked to multiple intermediaries affecting insulin signalling pathways, such as inhibiting insulin‐stimulated Tyr phosphorylation of IRS‐1 and subsequent recruitment and activation of PI3K [63], and activating PTP1B through the ceramide‐JNK‐NF‐κB signalling pathway, thereby inhibiting the phosphorylation of IRS‐1 and Akt, leading to insulin resistance [64, 65].

#### 3.1.2 Local Anaesthetic Agents

##### 3.1.2.1 Lidocaine

Lidocaine exhibits dose‐dependent effects on insulin secretion. At concentrations of 1–5 mM, it stimulates basal insulin release, but at 1 μM to 1 mM, it does not affect glucose‐induced (20 mM glucose) insulin secretion. Conversely, 5 mM lidocaine inhibits glucose‐stimulated insulin release [66]. When administered at therapeutic doses for treating muscle pain, lidocaine inhibits insulin‐stimulated glucose transport and glycogen synthesis. Studies show that lidocaine directly inhibits the insulin receptor (IR) kinase activity, decreasing insulin‐stimulated tyrosine phosphorylation of IR‐β (12.6% ± 5.7%, *p* < 0.001) and insulin receptor substrate (IRS)‐1 (32.1% ± 18.8%, *p* < 0.01), and disrupting the binding of IRS‐1 to the phosphoinositide 3‐kinase (PI3‐K) p85 regulatory subunit (27.4% ± 12.7%, *p* < 0.001) [67]. These findings illuminate lidocaine's molecular mechanism of inhibiting insulin signalling by suppressing IR tyrosine kinase activity and diminishing IRS‐1 phosphorylation and its interaction with PI3‐K.

##### 3.1.2.2 Bupivacaine

**FIGURE 1:**
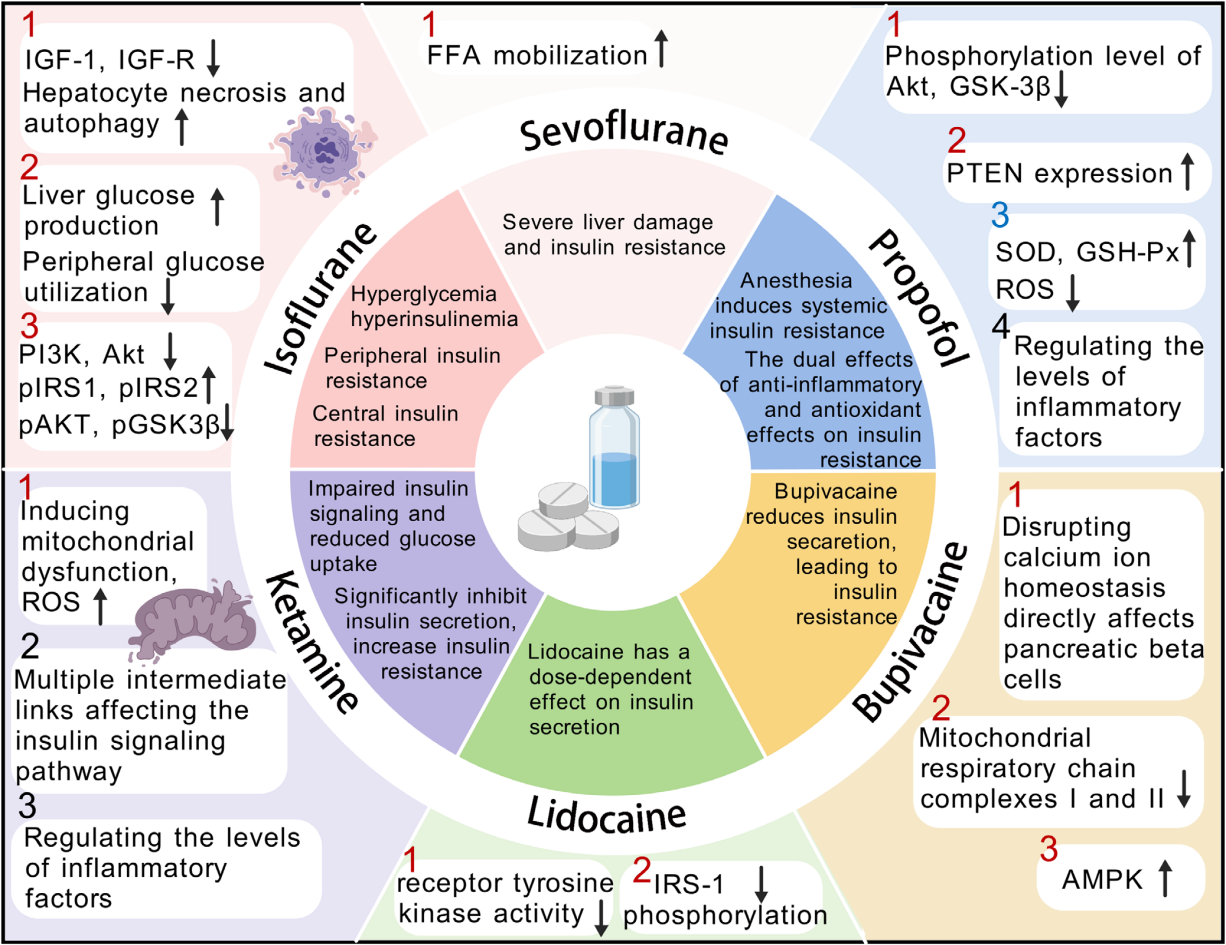
Impact of different Anaesthetics. This figure illustrates the effects of six anaesthetic agents on insulin resistance, with supporting references: Isoflurane induces hepatocyte necrosis via IGF‐1/IGF‐R downregulation [27] and alters insulin signalling through pIRS1/pIRS2 upregulation [32]. Sevoflurane promotes FFA mobilisation [34] and causes liver damage [37]. Propofol inhibits Akt/GSK‐3β [47] and activates SOD/GSH‐Px [49]. Ketamine induces mitochondrial dysfunction [58] and JNK activation [62]. Lidocaine inhibits IR kinase activity [67]. Bupivacaine impairs calcium homeostasis [68] and mitochondrial function [69]. Akt, protein kinase B; AMPK, adenosine monophosphate‐activated protein kinase; FFA, free fatty acid; GSH‐Px, glutathione peroxidase; GSK‐3β, Glycogen synthase kinase 3β; IGF‐1, insulin‐like growth factor 1; IGF‐R, insulin‐like growth factor receptor; IRS‐1, insulin receptor substrate 1; pAKT, phosphorylated protein kinase B; pGSK3β, phosphorylated glycogen synthase kinase 3β; PI3K, phosphatidylinositol 3‐kinase; pIRS1, phosphorylated insulin receptor substrate 1; pIRS2, phosphorylated insulin receptor substrate 2; PTEN, phosphatase and tensin homologue deleted on chromosome 10; ROS, reactive oxygen species; SOD, superoxide dismutase.

Bupivacaine may directly affect pancreatic β‐cells by disrupting calcium ion homeostasis, thereby inhibiting insulin secretion. Calcium ions are crucial for triggering insulin release, and bupivacaine blocks voltage‐gated calcium channels, reducing calcium influx and thus insulin secretion. Patients anaesthetised with bupivacaine may experience elevated blood glucose levels, more pronounced in diabetics. While bupivacaine inhibits insulin secretion, research on its direct impact on insulin resistance is limited. It is hypothesised that bupivacaine indirectly influences insulin signalling and energy metabolism. Bupivacaine inhibits mitochondrial respiratory chain complex I and II, leading to energy depletion and oxidative stress [68]. This oxidative stress suppresses the Akt/mTOR pathway and activates AMPK, contributing to insulin resistance [69]. Oxidative stress exacerbates insulin resistance by inhibiting insulin signalling pathways. Bupivacaine activates the AMPK signalling pathway, which is implicated in insulin resistance [70, 71]. The interplay between oxidative stress, the AMPK signalling pathway, and insulin resistance is complex and intertwined. In bupivacaine‐induced cardiotoxicity, AMPK activation reduces the inhibition of mTORC1 by phosphorylating TSC2 (tuberous sclerosis 2), leading to decreased activation of p70s6k and S6 [72, 73] (Figure 1). Bupivacaine reduces the phosphorylation of IRS1 (insulin receptor substrate 1) through feedback inhibition of p70s6k, thereby increasing the sensitivity of the insulin signalling pathway [74] (Figure 1). Intralipid (ILE) activates Akt in the insulin signalling pathway during recovery from bupivacaine toxicity, without affecting AMPK. This suggests that Intralipid may accelerate recovery from bupivacaine toxicity by modulating the insulin signalling pathway. Although there are abundant preclinical studies describing the effects of anaesthetics on metabolic pathways, bupivacaine lacks data from the human clinical field. Further research is needed in the future (Figure 1).

### 3.2 Impact on Weight and Lipid Metabolism

Anaesthesia impacts abdominal obesity indirectly, affecting fat metabolism pathways and altering fat distribution and storage. This can result in weight gain or uneven fat distribution post‐surgery. Anaesthesia also influences metabolic states, altering blood glucose and lipid levels. Different anaesthetic agents may cause varying changes in postoperative glucose and lipid levels, posing challenges in managing metabolic syndrome.

#### 3.2.1 Direct Effects on Lipid Metabolism

Anaesthetic agents directly influence lipid metabolism by affecting adipocyte differentiation. Propofol, for instance, affects peroxisome proliferator‐activated receptor γ (PPARγ) expression [75], promoting adipocyte differentiation and fat accumulation. Sevoflurane and isoflurane activate PPARα [76], enhancing hepatic fatty acid oxidation and reducing fatty acid accumulation. Research reveals that adipose tissue‐specific deletion of PPA1 causes severe lipodystrophy and insulin resistance. PPA1 maintains protein stability of transcription factors CEBP/β and CEBP/δ, regulating early adipocyte differentiation [77].

#### 3.2.2 Indirect Effects on Lipid Metabolism

Anaesthetics can activate the catecholamine system and induce transient insulin resistance, promoting fat mobilisation and breakdown. Propofol and fentanyl increase lipolytic enzyme activity [78], aiding pain management while impacting lipid metabolism. Local anaesthetics (LAs) like lidocaine, mepivacaine, ropivacaine and bupivacaine affect adipose stem cells (ASCs) in vitro. These drugs at 1–10 mM concentrations may not impact ASC survival but could reduce differentiation and secretion [77]. Recent research highlights lipophagy, a selective autophagy that degrades lipids, playing a crucial role in regulating cellular lipid metabolism and stability [79]. This process is modulated by genes, enzymes and transcriptional regulators. These findings underscore anaesthetics' importance in perioperative management and guide future clinical practices.

#### 3.2.3 Clinical Observations: Weight and Lipid Level Changes

Anaesthesia can alter postoperative lipid levels by affecting cholesterol synthesis and transport pathways, leading to changes in total cholesterol (TC) and low‐density lipoprotein cholesterol (LDL‐C) levels. Studies show that different propofol formulations affect perioperative lipid levels in hyperlipidemia patients. Propofol with medium‐chain fatty acids lowers LDL and TC levels significantly post‐surgery, with medium‐chain fatty acids showing greater reductions [80]. Propofol with long‐chain fatty acids has minimal impact on lipid levels, making it a safer choice for hyperlipidemia patients [81]. Propofol, known for its rapid induction and recovery, may increase triglyceride levels and liver dysfunction at higher doses, impacting hemodynamics. Highlighted clinical dose‐dependency: While animal studies show propofol improves insulin signalling via SOD activation [49], clinical studies indicate high‐dose propofol (> 4 mg/kg/h) increases triglyceride levels by 30% postoperatively in hyperlipidemic patients, revealing a dose‐dependent effect [81]. Sevoflurane in cardiac surgery reduces TC and LDL‐C [82], while fentanyl in general surgery decreases TC and high‐density lipoprotein cholesterol (HDL‐C) levels [83]. Propofol in orthopaedic surgery reduces TC and triglyceride levels [84]. These studies detail anaesthetics' impact on lipid levels, guiding perioperative patient management.

### 3.3 Impact on Blood Pressure

#### 3.3.1 General Anaesthetics

General anaesthetics regulate vascular tone by affecting smooth muscle contraction and endothelial‐mediated vasodilation. These agents dose‐dependently inhibit smooth muscle contraction by limiting Ca2+ influx, activating K+ channels and inhibiting contractile protein mechanisms. They also inhibit endothelial‐mediated vasodilation (NO, PGI2, EDHF). Inhalational anaesthetics like halothane, isoflurane and sevoflurane expand vascular smooth muscle by inhibiting Ca2+ influx, PKC, Rho kinase and p44/42 MAPK, and increasing KATP channel opening [85]. Etomidate minimally impacts hemodynamics, suitable for elderly, critically ill, shock and heart failure patients [86]. Ketamine excites the central sympathetic system, elevating blood pressure, heart rate and plasma catecholamine levels [87]. Midazolam's effects on respiratory and circulatory functions are dose‐dependent [88]. Dexmedetomidine reduces blood pressure and slows heart rate. Isoflurane may cause “coronary steal” in predisposed patients [89]. Inhalational anaesthetics dose‐dependently lower systemic blood pressure. Halothane and enflurane reduce stroke volume (SV) and cardiac output (CO) [90], while isoflurane, sevoflurane and desflurane maintain SV while decreasing systemic vascular resistance (SVR) [91].

#### 3.3.2 Local Anaesthetics

Local anaesthetics induce vasodilation, varying by drug and injection site, influenced by patient response. Vasodilation increases blood perfusion, speeding drug distribution, elevating plasma drug levels, shortening anaesthetic duration and increasing surgical bleeding. To counteract vasodilation, vasoconstrictors like epinephrine are added to local anaesthetics. This reduces local blood flow, slowing drug absorption and reducing systemic drug levels, enhancing neural blockade duration. Lidocaine, a common local anaesthetic, has rapid onset, strong action (1–2 h), strong penetration and wide safety margin. It inhibits neuronal Na^+^ channels, blocking nerve impulses [92]. Bupivacaine, a long‐acting local anaesthetic, is 45 times stronger and lasts 5–10 h, without significant vasodilation [93, 94]. Ropivacaine, a newer local anaesthetic, strongly blocks pain with minimal motor impact, shorter duration, lower cardiac toxicity than bupivacaine, and notable vasoconstriction. It inhibits neuronal Na^+^ channels and activates vascular smooth muscle α receptors, reducing blood perfusion, extending anaesthesia [95, 96]. These data highlight varying vascular effects of local anaesthetics and their clinical applications.

#### 3.3.3 Clinical Observations: Blood Pressure Changes in Metabolic Syndrome Patients

Metabolic syndrome patients exhibit significant blood pressure changes perioperatively, linked to their condition. Hypertension significantly increases metabolic syndrome risk, with risks 17 times higher in hypertensive groups. Anaesthesia‐induced myocardial inhibition and vasodilation can cause intraoperative hypotension, especially in diabetic and hypertensive patients. Studies show higher intraoperative hypotension rates in metabolic syndrome patients [97]. Sevoflurane use reduces mean arterial pressure (MAP) by about 15% intraoperatively and recovers postoperatively. A research indicated that the hemodynamic responses to thermoregulatory vasoconstriction were similar without anaesthesia and at all concentrations of desflurane and isoflurane. On average, mean arterial pressure increased 14 (SD = 5) mmHg with and without anaesthesia [98]. Moreover, another study compared the effects of dexmedetomidine, propofol and midazolam on mean arterial pressure (MAP) in sevoflurane‐anaesthetised rabbits. It was found that the MAP in the dexmedetomidine group was significantly lower than that in the control group and the midazolam group, and the heart rate (HR) was lower than that in all other groups [99]. In summary, anaesthetic drugs affect blood pressure and heart rate during surgery through various mechanisms. Clinical trials have also observed an increase in mean arterial pressure (MAP) values. Postoperative hypotension correlates with higher complication rates like acute kidney injury [100]. Long‐term blood pressure fluctuations increase stroke risk. Surgical interventions in metabolic syndrome patients improve weight, BMI, triglycerides, fasting glucose, and HbA1c and raise HDL and lowest oxygen saturation (LSaO2), reducing diabetes, hypertension, fatty liver and joint degeneration rates [101, 102]. These data emphasise the importance of postoperative blood pressure control in improving outcomes and reducing complications. However, human studies remain limited, and more clinical trials are needed for verification.

Table 1 systematically compares the effects of six common anaesthetic agents across four key MS‐related components.

**TABLE 1:** Effects of different anaesthetics on MS components.

Anaesthetic	Insulin resistance (Mechanism + evidence type)	Lipid metabolism (Mechanism + Evidence type)	Blood pressure (Mechanism + Evidence type)	Inflammation (Mechanism + Evidence type)
Isoflurane	Inhibits PI3K/Akt, increases pIRS1/pIRS2	Activates PPARα, enhances hepatic fatty acid oxidation	Inhibits Ca^2+^ influx, reduces systemic vascular resistance	Activates NF‐κB, increases TNF‐α
Sevoflurane	Promotes FFA mobilisation, reduces glucose uptake	Mildly increases triglycerides	Promotes NO release, reduces MAP by 15%	Inhibits NLRP3 inflammasome
Propofol	Inhibits Akt phosphorylation; High doses reduce inflammatory factors	Medium‐chain formulations lower LDL; High doses increase triglycerides	Inhibits sympathetic activity, reduces heart rate	Activates GABAR, decreases IL‐6
Ketamine	Inhibits insulin secretion, activates JNK pathway	Affects adipocyte differentiation	Stimulates sympathetic nervous system, increases blood pressure	Inhibits M1 macrophage inflammation
Lidocaine	High concentrations inhibit IR tyrosine kinase	Impairs adipose stem cell differentiation	Causes vasodilation, increases local perfusion	Inhibits NF‐κB
Bupivacaine	Disrupts calcium homeostasis, reduces insulin secretion	Inhibits mitochondrial complexes, induces oxidative stress (In vitro)	No significant vasodilation	Activates AMPK, indirectly modulates inflammation

### 3.4 Postoperative Progression of Metabolic Syndrome and Anaesthetic Implications

The metabolism and excretion of anaesthetic agents can influence postoperative metabolic homeostasis and recovery kinetics. Additionally, anaesthesia may indirectly exacerbate metabolic syndrome symptoms by modulating postoperative pain management and inflammatory responses, with potential long‐term consequences for patient recovery and overall health.

#### 3.4.1 Mechanistic Insights

Multiple interrelated factors contribute to the aggravation of metabolic syndrome during postoperative recovery, including systemic inflammation, endocrine dysregulation, nutritional deficits, suboptimal analgesia and psychological stress. Surgical trauma triggers a pronounced inflammatory response, with elevated interleukin‐6 (IL‐6) levels correlating strongly with metabolic deterioration in non‐cardiac surgical patients (*r* = 0.42, *p* < 0.01) [103]. Concurrently, perioperative endocrine perturbations—notably insulin resistance and hypercortisolemia—drive metabolic dysregulation. Cortisol levels peak within 24 h postoperatively, directly stimulating lipolysis and hyperglycemia (Δcortisol: +58%, *p* = 0.003 vs. baseline) [104].

Protein‐calorie malnutrition exacerbates catabolism and insulin insensitivity, particularly in elderly surgical cohorts, where inadequate nutritional intake within 72 h postoperation amplifies metabolic syndrome severity (OR 2.1, 95% CI 1.4–3.2) [105]. Analgesic strategies further modulate outcomes: multimodal regimens combining NSAIDs and regional anaesthesia attenuate stress hormone release more effectively than opioid‐centric protocols (postoperative glucose: 128 ± 18 mg/dL vs. 154 ± 22 mg/dL, *p* = 0.02). Psychological comorbidities compound these effects, with anxiety/depression correlating with heightened inflammatory markers (CRP: +32%, *p* = 0.01) and worsened metabolic parameters [106].

## 4 Impact of Metabolic Syndrome on Anaesthesia Drugs

Metabolic syndrome significantly influences the metabolism and efficacy of anaesthesia drugs, potentially reshaping the overall strategy for anaesthesia management. This section delves into the primary impacts of metabolic syndrome on anaesthesia drugs, focusing on drug metabolism and clearance rates.

### 4.1 Drug Metabolism

Metabolic syndrome can alter the activity of drug‐metabolising enzymes, thus modifying the metabolism of anaesthesia drugs. For instance, the chronic inflammatory state and hepatic fat accumulation often associated with metabolic syndrome can impair hepatic enzyme function. Given that most anaesthesia drugs are metabolised in the liver, such changes can affect their efficacy and side effects by altering their concentrations in the body.

### 4.2 Insulin Resistance and Drug Metabolism

#### 4.2.1 Drug Metabolic Enzyme

Metabolic syndrome impacts drug metabolising enzymes, particularly the cytochrome P450 (CYP450) enzyme system, through intricate mechanisms encompassing gene expression regulation, microRNA modulation, inflammatory mediators, protein synthesis and degradation, oxidative stress, cellular apoptosis and necrosis and tissue repair processes [107]. For example, inflammation and oxidative stress induced by metabolic syndrome can influence the transcription of genes involved in CYP450 enzyme expression and modulate the expression of certain microRNAs, such as miR‐33, which downregulates CYP450 enzymes by inhibiting SREBP‐1 [108, 109]. Additionally, adipose tissue accumulation leads to the release of adipokines and inflammatory mediators like TNF‐α and IL‐6, which can directly inhibit CYP450 enzyme expression and activity [110]. High glucose levels and oxidative stress impact protein synthesis and degradation, leading to the accelerated formation of advanced glycation end‐products (AGEs) that can degrade specific CYP450 enzyme subtypes or cause structural changes reducing their catalytic efficiency [111]. Oxidative stress not only directly damages CYP450 enzymes but also influences their activity through changes in the NADPH/NADP+ ratio [112]. These mechanisms are crucial for understanding drug metabolism, enabling the development of personalised medication plans, predicting and preventing drug interactions and optimising drug development to ensure efficacy and safety in metabolic syndrome patients.

#### 4.2.2 Drug Clearance

Metabolic syndrome significantly impacts the clearance rate and half‐life of anaesthesia drugs, primarily through its effects on liver and kidney function and the CYP450 enzyme system. For example, metabolic syndrome patients often have hypertension and cardiovascular diseases, which can reduce hepatic blood flow and thus decrease the clearance rate of anaesthesia drugs [113]. Additionally, due to inflammation and oxidative stress, hepatic enzyme activity in metabolic syndrome patients may decrease, affecting drug metabolism [114]. In terms of renal function, metabolic syndrome patients frequently have diabetes and hypertension, which can lead to renal insufficiency, affecting the clearance of anaesthesia drugs dependent on renal excretion, such as remifentanil, which can have its half‐life extended in metabolic syndrome patients [115, 116]. Metabolic syndrome significantly impacts the clearance rate and half‐life of anaesthesia drugs [117], and clinicians need to consider these factors when selecting anaesthetic drugs for metabolic syndrome patients to ensure anaesthesia safety and efficacy.

#### 4.2.3 Impact of Body Weight and Lipid Metabolism on Medications

##### 4.2.3.1 Adipose Tissue's Role

Adipose tissue plays a crucial role in the distribution and metabolism of anaesthetic drugs, influenced by its volume and distribution and interconnected metabolic mechanisms. Fat tissue, capable of storing substantial amounts of drugs, can lead to higher drug concentrations therein compared to other tissues. Consequently, patients with abundant adipose tissue may experience prolonged retention of drugs within these tissues, affecting the onset and metabolism rate of the drugs [118]. Studies indicate that obese patients (BMI ≥ 30 kg/m^2^) exhibit significantly increased volume of distribution (Vd) for lipophilic drugs [119]. Moreover, adipocytes, besides functioning as energy storage organs, participate in various metabolic activities, including drug metabolism [120]. Additionally, uneven blood flow distribution within adipose tissue can affect drug transport and distribution, potentially leading to regional variations in drug absorption and metabolism [121, 122]. Moreover, adipose tissue not only participates in energy metabolism, but also has immunomodulatory functions. Immune cells in adipose tissue can influence the expression and activity of drug‐metabolising enzymes. Changes in immunomodulatory function may lead to changes in the rate and effects of drug metabolism, especially in patients with obesity and metabolic syndrome. Studies have shown that macrophages in the adipose tissue of obese patients increase in numbers and that these cells can secrete multiple inflammatory factors that affect the expression and activity of drug‐metabolising enzymes [123]. For example, TNF‐α secreted by macrophages can induce the expression of hepatic cytochrome P450 enzyme, affecting drug metabolism [124, 125].

### 4.3 Intraoperative Drug Requirements

Patients with metabolic syndrome may experience altered effects of anaesthetic drugs, encompassing analgesic efficacy, depth of anaesthesia and side effects.

#### 4.3.1 Dose Titration

Adjusting dosages for these patients is crucial, considering their unique physiological states such as obesity, hypertension, diabetes and hyperlipidemia, significantly affecting the pharmacokinetics and pharmacodynamics of anaesthetic drugs [126]. For instance, the volume of distribution for propofol increases in obese and metabolic syndrome patients, resulting in lower plasma concentrations and prolonged maintenance times. Research suggests a possible 10%–15% reduction in propofol clearance rates in obese patients, necessitating increased doses to achieve the desired anaesthesia depth and duration [127]. Similarly, reductions in etomidate metabolism rates in metabolic syndrome patients warrant increased doses to ensure expected anaesthesia effects [128, 129]. Notably, the half‐life of midazolam may extend by about 20% in these patients, with reduced clearance rates, necessitating initial dose reductions to prevent drug accumulation and excessive sedation [130]. Furthermore, the half‐life of remifentanil, dependent on renal clearance, can extend by 25%–30% in metabolic syndrome patients, leading to reduced doses to avoid excessive accumulation and prolonged analgesia [131, 132]. Therefore, individualised dose adjustment according to the metabolic syndrome status is needed to ensure the optimal analgesic effect.

#### 4.3.2 Side Effects

Metabolic syndrome patients undergoing anaesthesia face increased risks of adverse effects and complications. Obesity, limiting thoracic and abdominal movement and hindering diaphragm descent, reduces lung capacity and increases postoperative respiratory complications, including hypoxemia, hypercapnia, dyspnea and respiratory depression [133]. Hypertensive patients face heightened risks of blood pressure fluctuations, increasing risks of myocardial ischemia and cerebrovascular accidents, manifesting as arrhythmias, myocardial ischemia, infarctions and cerebrovascular accidents [134]. High glucose levels can impact drug metabolism, increasing postoperative infection and poor wound healing risks, resulting in intraoperative hyperglycemia, postoperative infections and delayed wound healing [133]. Higher analgesic doses may be required for obese patients, and diabetic patients with peripheral neuropathy may necessitate more robust analgesic management, resulting in intensified postoperative pain and inadequate analgesic effects. Postoperative risks for metabolic syndrome patients include increased risks of respiratory, cardiovascular and wound healing complications, manifesting as respiratory difficulties, cardiovascular events, wound infections and dehiscence [135].

### 4.4 Postoperative Recovery

Metabolic syndrome can significantly impact postoperative recovery processes, including pain management and overall recovery speed.

#### 4.4.1 Resumption Period Characteristics

Effective pain management is particularly critical for these patients, given the higher incidence of pain and the need for comprehensive pain control strategies [136]. Nutritional support is also vital during this period, as rapid weight loss and inadequate nutrient intake can lead to deficiencies in vitamins and minerals, particularly thiamine (Vitamin B1) [137, 138]. Thromboprophylaxis is essential due to heightened postoperative thrombosis risks, with recommendations for low molecular weight heparin (LMWH) for patients with a BMI ≥ 40 kg/m^2^ [139]. Preventing gastroesophageal reflux and gastric ulcers, especially post‐gastric bypass surgery (RYGB), is necessary, with proton pump inhibitors (PPIs) significantly reducing the risk of marginal ulcers [140]. Adequate protein and energy intake, particularly for patients undergoing restrictive or malabsorptive surgeries, is crucial to prevent protein‐energy malnutrition [141]. Additionally, the risks of deficiencies in iron, folate, vitamin B12, vitamin D and trace elements such as zinc, copper and selenium are heightened, along with increased risks for vitamins A, E and K in malabsorptive surgical cases.

In conclusion, individualised management strategies and high‐standard postoperative care are key to promoting recovery in metabolic syndrome patients, requiring adjustments based on individual metabolic conditions and specific needs. Clinical data support these strategies, providing effective management evidence.

### 4.5 Clinical Management Strategies

#### 4.5.1 Individualised Anaesthesia Plan

An individualised anaesthesia plan is crucial for ensuring the safety of patients with metabolic syndrome during and after surgery. The unique physiological conditions of these patients can lead to variations in the metabolism and response to anaesthetic drugs [142]. Factors such as obesity and impaired liver function can affect the distribution, metabolism and excretion of drugs, thereby increasing the risk of side effects. Therefore, adjusting the selection and dosage of anaesthetic drugs based on the specific conditions of the patient is essential. Obese patients may require higher drug doses, while those with liver dysfunction may need reduced doses or drugs that have minimal impact on the liver [143].

Using a single anaesthetic drug may not adequately address the specific needs of metabolic syndrome patients and could increase the risk of side effects. Consequently, a multimodal approach to anaesthesia is particularly important. This strategy involves the combined use of different types of anaesthetic drugs, such as local anaesthesia, regional anaesthesia and general anaesthesia [144]. This combination can enhance pain management, reduce the dose of each drug and minimise side effects. Selecting the optimal combination of these anaesthetic methods based on the type of surgery and the patient's condition is an effective approach to managing anaesthesia in metabolic syndrome patients.

During surgery, the response of metabolic syndrome patients to anaesthetic drugs may vary significantly, necessitating close monitoring of drug effects and side effects. Potential issues include excessive or insufficient anaesthesia, as well as drug side effects such as respiratory depression and hypotension. Advanced monitoring equipment, such as bispectral index (BIS) to monitor anaesthesia depth, and continuous monitoring of blood pressure, heart rate and blood oxygen saturation, are crucial for ensuring patient safety [145]. Adjusting the type and dosage of anaesthetic drugs in a timely manner can help maintain optimal conditions for the patient.

Additionally, metabolic syndrome patients may face a higher risk of complications during the anaesthesia recovery period, requiring special attention to drug clearance and management. Continuous monitoring of drug metabolism and clearance post‐operatively, adjusting the frequency and dosage of drug use, and ensuring a safe recovery are key components of anaesthesia recovery management.

In summary, an individualised anaesthesia plan is vital for ensuring the safety of metabolic syndrome patients during and after surgery. By adjusting the selection and dosage of anaesthetic drugs based on the patient's specific conditions, implementing a multimodal anaesthesia strategy, and intensifying intraoperative monitoring and management, we can enhance patient safety and recovery. Clinical data support these strategies and provide evidence of their effectiveness.

## 5 Mechanistic Exploration of Bidirectional Relationships

### 5.1 Insulin Signalling Pathway

#### 5.1.1 Anaesthetic Drug Influence

General anaesthesia can induce dysfunction in the insulin receptor (IR) signalling pathway, thereby affecting the biological effects of insulin [146]. Studies have shown that general anaesthesia can significantly elevate blood glucose levels in diabetic patients, increase insulin secretion, yet decrease the glucose‐to‐insulin (G/I) ratio, indicating the development of insulin resistance [147]. Moreover, general anaesthetic agents may directly impact the PI3K/Akt signalling pathway, thereby altering insulin sensitivity.

Research suggests that insulin in the brain may inhibit inflammation induced by amyloid‐beta (Aβ) [148]. However, abnormalities in the insulin signalling pathway can exacerbate Aβ‐induced inflammatory responses [149]. Aβ can induce phosphorylation at multiple sites of insulin receptor substrate‐1 (IRS‐1), such as Ser312 and Ser612, leading to inhibition of the PI3K/Akt pathway in the insulin signalling cascade [150].

**FIGURE 2:**
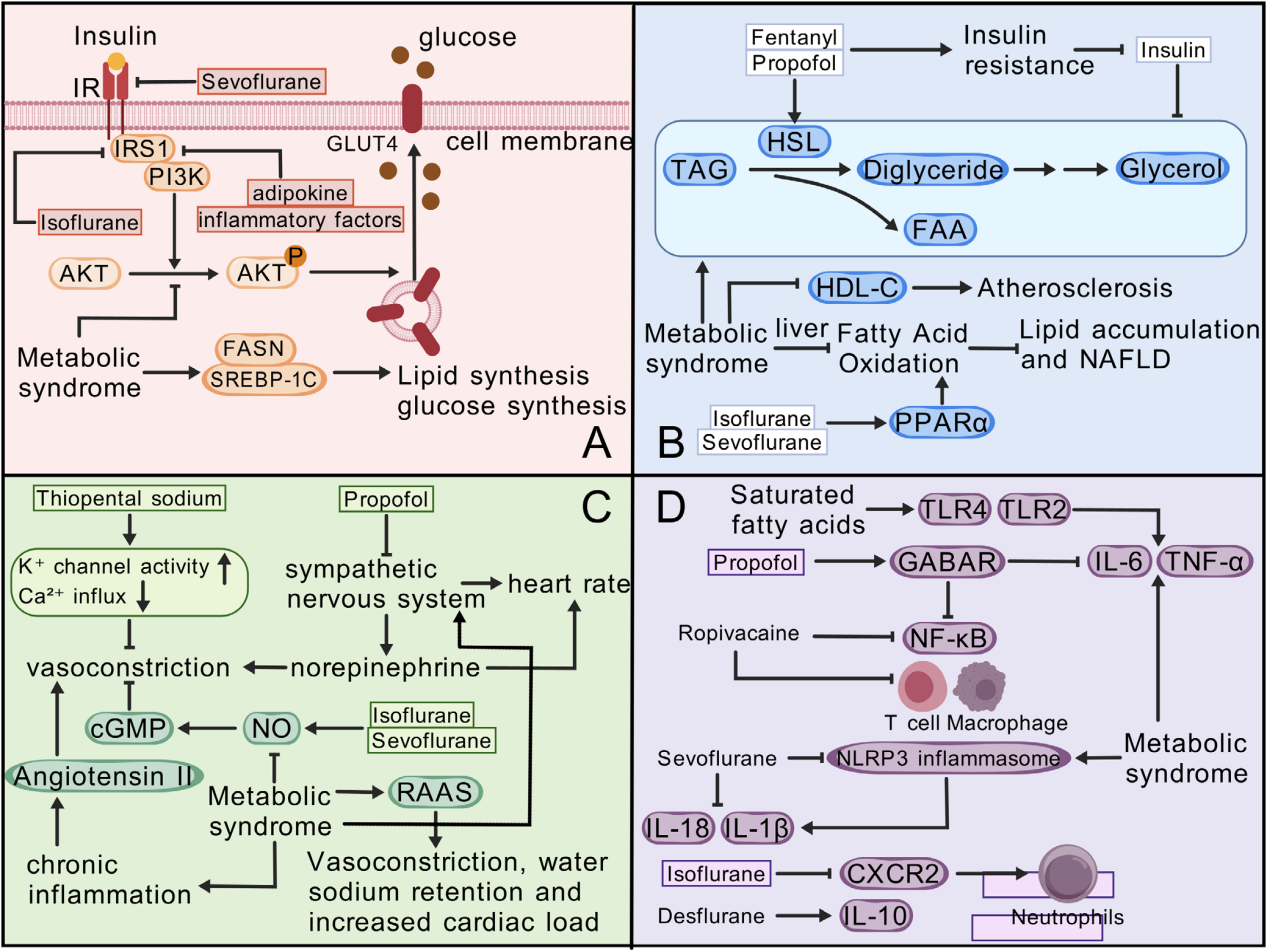
Mechanistic Exploration of Bidirectional Relationships. This figure illustrates the bidirectional effects of different anaesthetic agents on pathways related to metabolic syndrome. (A) After insulin binds to the insulin receptor (IR), sevoflurane and isoflurane inhibit glucose transport into the cells via the IRS‐1, PI3K, AKT signalling pathways [32, 38]. Metabolic syndrome also inhibits glucose transport by suppressing AKT phosphorylation, while influencing lipid and glucose synthesis through FASN and SREBP‐1C [152]. (B) Fentanyl and propofol affect triglyceride (TAG) metabolism by activating hormone‐sensitive lipase (HSL), leading to insulin resistance. Metabolic syndrome promotes free fatty acid (FAA) generation, inhibits high‐density lipoprotein cholesterol (HDL‐C) production, and impairs liver fatty acid oxidation, which is associated with atherosclerosis, lipid accumulation and non‐alcoholic fatty liver disease (NAFLD) [78]. Isoflurane and sevoflurane act on PPARα to enhance fatty acid oxidation and reduce liver lipid accumulation [153]. (C) Thiopental affects potassium channel activity and calcium ion influx, leading to vasodilation. Propofol influences the sympathetic nervous system by inhibiting norepinephrine release, promoting vasodilation and lowering heart rate. Isoflurane and sevoflurane inhibit vasoconstriction by promoting nitric oxide (NO) and cyclic GMP (cGMP). Metabolic syndrome acts on NO and angiotensin II to promote vasoconstriction [184; 188], while also excessively activating the renin‐angiotensin‐aldosterone system (RAAS), causing water and sodium retention, increased cardiac load and chronic inflammation [154]. (D) Propofol inhibits the release of pro‐inflammatory factors such as IL‐6 and TNF‐α by activating γ‐aminobutyric acid A receptor (GABAR). Ropivacaine suppresses the NF‐κB pathway and the functions of T cells and macrophages. Sevoflurane inhibits the NLRP3 inflammasome, suppressing the release of IL‐1β and IL‐18. Isoflurane inhibits neutrophils through CXCR2, and desflurane promotes the generation of the anti‐inflammatory factor IL‐10 [155]. Metabolic syndrome promotes the NLRP3 inflammasome and the release of pro‐inflammatory factors like IL‐6 and TNF‐α. Saturated fatty acids activate TLR2 and TLR4, promoting the synthesis of pro‐inflammatory cytokines such as TNF‐α and IL‐6 [156]. AKT, protein kinase B; cGMP, cyclic guanosine monophosphate; CXCR2, CXC‐chemokine receptor 2; FAA, free fatty acid; FASN, fatty acid synthase; GABAR, γ‐aminobutyric acid A receptor; GLUT4, glucose transporter 4; HDL‐C, high‐density lipoprotein cholesterol; HSL, hormone‐sensitive lipase; IL‐6, interleukin‐6; IL‐10, interleukin‐10; IL‐18, interleukin‐18; IL‐1β, interleukin‐1β; IR, insulin receptor; IRS‐1, insulin receptor substrate‐1; NAFLD, non‐alcoholic fatty liver disease; NF‐κB, nuclear factor‐κB; NLRP3, NOD‐like receptor protein 3; NO, nitric oxide; PI3K, phosphatidylinositol 3‐kinase; PPARα, peroxisome proliferator‐activated receptor α; RAAS, Renin‐Angiotensin‐Aldosterone System; SREBP‐1C, sterol‐regulatory element binding protein‐1C; TAG, triglyceride; TLR2, toll‐like receptor 2; TLR4, toll‐like receptor 4; TNF‐α, tumour necrosis factor‐α.

The proximal events of the insulin signalling pathway include the activation of the insulin receptor (IR) and the phosphorylation of signalling proteins, particularly IRS, PI3K and Akt isoforms [151]. These events are highly conserved in insulin target tissues and initiate insulin responses on the plasma membrane. The PI3K‐dependent pathway mediates glucose, lipid and protein metabolism, as well as insulin‐stimulated glucose uptake (Figure 2A). Upon activation, Akt phosphorylates various downstream substrates in skeletal muscle, adipose tissue and the liver, facilitating the retention of insulin‐mediated nutrients in these tissues [157].

Moreover, isoflurane can activate protein kinase C (PKC), increasing the serine phosphorylation of IRS‐1, thereby inhibiting its function and reducing insulin signalling [158, 159]. Isoflurane can also activate the nuclear factor‐kappa B (NF‐κB) pathway, increasing the expression of pro‐inflammatory factors such as tumour necrosis factor‐alpha (TNF‐α), further suppressing the insulin signalling pathway [160] (Figure 2A). Additionally, sevoflurane inhibits the autophosphorylation of the insulin receptor, reducing the activation of downstream signalling molecules and thereby decreasing the efficiency of insulin signal transduction [161]. Sevoflurane also activates 5′‐AMP‐activated protein kinase (AMPK), inhibiting the mTOR signalling pathway and consequently reducing glucose uptake and utilisation [162]. Propofol inhibits the phosphatidylinositol 3‐kinase (PI3K)/protein kinase B (Akt) pathway, reducing the translocation of insulin‐stimulated glucose transporter 4 (GLUT4), thereby inhibiting glucose uptake [163]. In clinical practice, the metabolic effects of these anaesthetic agents should be considered, especially in diabetic or metabolic syndrome patients.

#### 5.1.2 Metabolic Syndrome Influence

Metabolic syndrome (MetS) is often accompanied by insulin resistance, a condition that impairs insulin signalling pathways [164]. The liver and skeletal muscle are key sites for insulin signalling. In individuals with MetS, hepatic steatosis and inflammation lead to insulin resistance in hepatocytes, primarily through increased expression of SREBP‐1c and FASN, which promote lipid synthesis and gluconeogenesis [152, 165] (Figure 2A). In skeletal muscle, insulin signalling abnormalities are characterised by reduced Akt activation, resulting in decreased GLUT4 translocation and glucose uptake. MetS‐induced muscle insulin resistance is also associated with a shift in muscle fibre type from oxidative to glycolytic and a reduction in mitochondrial content [166, 167]. This shift diminishes insulin sensitivity in muscle cells, mainly by reducing GLUT4 expression and translocation, thereby decreasing glucose uptake. Additionally, lactate accumulation in glycolytic fibres can indirectly impair insulin signalling by inhibiting Akt phosphorylation [168].

Adipose tissue is another critical site for insulin signalling. MetS can disrupt insulin signalling pathways through increased inflammation and adipose tissue mass, reducing insulin sensitivity. In the context of a high‐fat diet and obesity, downregulation of the insulin receptor and inhibition of signal transduction can significantly affect insulin efficacy. Research indicates that inflammation in the adipose tissue of MetS patients increases the production of adipokines [169], which interfere with IRS‐1 signalling, blocking the PI3K‐Akt pathway and inhibiting GLUT4 translocation, leading to insulin resistance [170] (Figure 2A). Aberrant expression of FABP4 and FFAR2/3 is also closely linked to insulin resistance in MetS [171].

In MetS, adipose tissue dysfunction extends beyond inflammation to include reduced activity of brown adipose tissue and lipid accumulation in white adipose tissue [172]. These changes impair mitochondrial function and reduce AMPK activity, affecting key molecules in insulin signalling such as GLUT4 and PDK1 [173]. Moreover, adipocyte hypertrophy and hyperplasia in MetS lead to increased secretion of plasminogen activator inhibitor‐1 (PAI‐1), further exacerbating insulin resistance. Chronic low‐grade inflammation, a hallmark of MetS, plays a significant role in insulin resistance. Inflammatory cytokines such as CRP, IL‐6 and TNF‐α activate JNK and IKKβ, increasing serine phosphorylation and reducing tyrosine phosphorylation of IRS‐1, thereby inhibiting insulin signalling [170] (Figure 2A). Downregulation of the PI3K subunit p85α, mediated by inflammation, is another important mechanism of insulin resistance [174, 175].

Through these mechanisms, metabolic syndrome significantly affects insulin signal transduction, leading to systemic insulin resistance, thereby influencing glucose and lipid metabolism and increasing the risk of cardiovascular disease. These findings not only elucidate the pathogenesis of MetS but also provide a theoretical basis for the development of new therapeutic strategies.

### 5.2 Lipid Metabolism

#### 5.2.1 Anaesthesia Agents and Their Impact on Lipid Metabolism

Anaesthesia agents exert multifaceted effects on lipid metabolism, particularly through the regulation of lipolysis and fat mobilisation. Certain agents, such as Propofol and Fentanyl, enhance the activity of hormone‐sensitive lipase (HSL), leading to the hydrolysis of stored triglycerides (TAG) into free fatty acids (FFA) and glycerol [176] (Figure 2B). This is mediated via the activation of adrenergic receptors, which increase cyclic AMP (cAMP) levels. Additionally, these agents may induce transient insulin resistance, thereby reducing the inhibitory effect of insulin on adipocyte lipolysis. Insulin normally promotes glucose uptake and TAG synthesis, while inhibiting TAG hydrolysis and FFA release [177] (Figure 2B). However, anaesthesia‐induced alterations in the phosphatidylinositol 3‐kinase (PI3K) pathway through PTEN and SHIP proteins disrupt insulin signalling, promoting lipid release [178]. Furthermore, anaesthesia agents can influence hepatic lipid metabolism pathways, such as fatty acid synthesis and oxidation, affecting serum lipid levels. For instance, agents like Sevoflurane activate PPARα, enhancing fatty acid oxidation and reducing hepatic lipid accumulation [179] (Figure 2B). Understanding these mechanisms is crucial for managing postoperative lipid metabolism and reducing metabolic complications.

#### 5.2.2 Metabolic Syndrome and Its Impact on Lipid Metabolism

Metabolic syndrome (MetS) significantly disrupts lipid metabolism, often associated with high cholesterol and triglyceride levels, increasing cardiovascular risk. Central obesity, a hallmark of MetS, exacerbates adipose tissue lipolysis, leading to elevated FFA levels [180]. Concurrently, hepatic fatty acid oxidation is impaired, causing lipid accumulation and non‐alcoholic fatty liver disease (NAFLD) [153] (Figure 2B). High triglyceride levels, driven by dietary fats, carbohydrate intake and increased hepatic TAG synthesis via SREBP‐1c and VLDL production, contribute to hypertriglyceridemia [181]. Reduced high‐density lipoprotein cholesterol (HDL‐C) in MetS impairs cholesterol reverse transport, enhancing atherogenesis (Figure 2B). Dysregulated lipid metabolism in MetS can influence anaesthesia dynamics, potentially altering drug pharmacokinetics and efficacy [182, 183]. This necessitates meticulous monitoring and tailored anaesthesia management in MetS patients.

### 5.3 Blood Pressure Regulation

#### 5.3.1 Anaesthesia Agents and Their Impact on Blood Pressure Regulation

Anaesthetic agents modulate blood pressure regulation primarily through vasodilation and modulation of the sympathetic nervous system. The inhibition of vascular smooth muscle (VSM) contraction by anaesthetics is a key mechanism underlying vasodilation and reduced effective circulating blood volume [184]. Anaesthetics dose‐dependently inhibit VSM contraction by restricting Ca^2+^ influx [185] and sarcoplasmic reticulum Ca^2+^ release, activating K^+^ channels [186] and inhibiting the sensitisation and phosphorylation of contractile proteins [187] (Figure 2C).

Thiopental sodium induces vasodilation by decreasing Ca^2+^ influx and sarcoplasmic reticulum Ca^2+^ release, increasing K^+^ channel activity and inhibiting the sensitisation and phosphorylation of contractile proteins. General anaesthetics can also suppress sympathetic nervous system activity, reducing norepinephrine release [188] or promoting endothelial nitric oxide (NO) release [189], and increasing intracellular cyclic guanosine monophosphate (cGMP) levels to activate cGMP‐dependent protein kinase [190], leading to significant vasodilation (Figure 2C).

Propofol lowers blood pressure by inhibiting norepinephrine (NE) release, reducing sympathetic nervous system excitability, and alleviating vasoconstriction and tachycardia [191] (Figure 2C). Sevoflurane promotes endothelial NO release, activates guanylate cyclase and increases intracellular cGMP levels to induce vasodilation and lower blood pressure. Isoflurane increases cGMP levels and activates cGMP‐dependent protein kinase, resulting in VSM relaxation and vasodilation [192].

#### 5.3.2 Metabolic Syndrome and Its Impact on Blood Pressure Regulation

Metabolic syndrome (MetS) significantly impacts blood pressure regulation, primarily through insulin resistance, chronic low‐grade inflammation and sympathetic nervous system overactivity. Insulin resistance impairs insulin signalling, leading to reduced NO production and vascular smooth muscle dysfunction [193]. Chronic inflammation in MetS exacerbates vascular damage and elevates blood pressure via increased expression of vasoconstrictors like angiotensin II [194] (Figure 2C). In MetS, chronic inflammation and vascular damage are associated with the persistent activation of the MAPK pathway [194], which regulates cell proliferation, differentiation and inflammation, leading to vasoconstriction and elevated blood pressure. Oxidative stress and lipid peroxidation products, such as 4‐hydroxynonenal (4‐HNE) and malondialdehyde (MDA) [195], activate NF‐κB and the NLRP3 inflammasome in MetS, further enhancing inflammatory responses and vascular damage, resulting in increased blood pressure [196]. The renin‐angiotensin‐aldosterone system (RAAS) is also hyperactive in MetS, contributing to vasoconstriction, water and sodium retention and increased cardiac workload [197]. Sympathetic overactivity in MetS further elevates heart rate, vascular tone and blood pressure [154] (Figure 2C). These factors necessitate close monitoring and adaptive anaesthesia management in MetS patients to prevent adverse cardiovascular events.

### 5.4 Inflammatory Response

#### 5.4.1 Anaesthesia Agents and Their Impact on Systemic Inflammatory Response

Anaesthesia agents exert significant effects on the systemic inflammatory response, primarily through anti‐inflammatory mechanisms [198]. These agents inhibit the release of proinflammatory cytokines such as TNF‐α and IL‐6, thereby mitigating systemic inflammation [199]. For example, Propofol activates GABA receptors, inhibiting the release of proinflammatory cytokines and reducing NF‐κB activation, which lowers inflammatory mediator production [155] (Figure 2D). Sevoflurane inhibits the activation of the NLRP3 inflammasome, reducing the release of IL‐1β and IL‐18 and modulates caspase‐1 activity to decrease inflammasome activation [200] (Figure 2D). Ropivacaine inhibits macrophage and T‐cell functions, reducing inflammatory mediator production and inhibiting NF‐κB activation [201] (Figure 2D). Desflurane activates endogenous anti‐inflammatory mechanisms, such as increasing IL‐10 production, enhancing the anti‐inflammatory capacity of the body [202]. Anaesthesia agents also influence immune cell functions, such as Isoflurane inhibiting neutrophil inflammatory responses by interfering with the CXCR‐2 chemokine receptor [203] (Figure 2D). Understanding these mechanisms is crucial for managing perioperative inflammation and enhancing patient outcomes.

#### 5.4.2 Metabolic Syndrome and Its Impact on Inflammatory Response

Metabolic syndrome (MetS) is characterised by a complex interplay of factors that contribute to chronic low‐grade inflammation [204]. This inflammatory state significantly impacts systemic inflammatory responses, influencing the efficacy of anaesthetic agents [205]. Mechanisms include elevated levels of proinflammatory cytokines like TNF‐α and IL‐6, which activate inflammatory signalling pathways [206] and disrupt insulin signalling. NLRP3 inflammasome activation, influenced by metabolic byproducts in obese individuals, promotes the release of IL‐1β and IL‐18, further exacerbating inflammation [207] (Figure 2D). Saturated fatty acids activate TLR2 and TLR4, promoting the synthesis of proinflammatory cytokines like TNF‐α, IL‐6 and MCP‐1 [156] (Figure 2D). Additionally, long‐term energy surplus leads to TAG deposition in adipocytes [208], inducing inflammation and insulin resistance. Immune cells in adipose tissue, including lymphocytes, NK cells, mast cells and dendritic cells, play crucial roles in MetS development, regulating inflammation and immune function [209] (Figure 2D). This underscores the importance of managing inflammation in MetS patients during anaesthesia to enhance perioperative outcomes.

## 6 Optimising Anaesthesia Protocols

With a deepening understanding of the mechanisms underlying the interaction between metabolic syndrome and anaesthetic agents, future research should focus on optimising anaesthesia protocols to enhance both therapeutic efficacy and safety. Personalised anaesthesia emerges as a pivotal direction, particularly for patients with metabolic syndrome. Personalised anaesthesia regimens should be tailored based on the specific metabolic condition, disease stage and other health metrics of the patient, adjusting both the selection and dosage of anaesthetic agents [210]. This approach can mitigate drug side effects, thereby improving the overall safety and efficacy of anaesthesia [211, 212].

For patients with metabolic syndrome, the adjustment of drug selection and dosage is especially critical [213]. The choice of anaesthetic agents should be based on the patient's metabolic profile, such as insulin sensitivity, lipid metabolism status and blood pressure levels. Proper dosage adjustments can reduce intraoperative and postoperative complications, enhancing the effectiveness of anaesthesia [214]. For instance, in patients with insulin resistance, selecting anaesthetics with minimal metabolic interference or adjusting dosages to avoid adverse reactions like hyperglycemia represents a strategic approach to optimising anaesthesia protocols [31, 215].

### 6.1 Postoperative Management

Postoperative management is equally crucial for the recovery of patients with metabolic syndrome [216]. Strategies to improve postoperative weight management, blood sugar control and lipid level adjustments significantly impact overall recovery and overall health [217, 218].

Effective postoperative weight management can be achieved through the implementation of a balanced dietary plan and an exercise regimen, which help reduce fat accumulation and alleviate symptoms of metabolic syndrome [9, 218, 219]. Blood sugar control is another critical area, with stable postoperative blood sugar levels being particularly important for metabolic syndrome patients. By closely monitoring blood sugar levels and adjusting insulin or other hypoglycemic agents as necessary, postoperative blood sugar fluctuations can be effectively controlled [220]. Moreover, lipid level adjustments are equally important, especially for patients with high cholesterol or triglyceride levels. Through pharmacological treatment and lifestyle interventions, lipid levels can be managed, thereby reducing the risk of postoperative complications [9].

### 6.2 Potential of Novel Anaesthetics

Research into novel anaesthetic agents represents a significant future direction. Currently, many traditional anaesthetics may have adverse effects on patients with metabolic syndrome [9], making the exploration of metabolic syndrome‐friendly novel anaesthetics essential. These new agents should possess superior metabolic characteristics, fewer side effects and higher safety, effectively reducing adverse impacts on metabolic syndrome patients. Preclinical and clinical trials to develop and test these new drugs will provide more therapeutic options for metabolic syndrome patients. Through these studies, more scientific and practical guidelines can be provided for clinical practice, improving the anaesthesia management and postoperative recovery of metabolic syndrome patients.

In summary, future research and clinical applications should focus on optimising anaesthesia protocols, enhancing postoperative management and exploring novel anaesthetic drugs to address the challenges posed by metabolic syndrome, thereby improving therapeutic outcomes and quality of life for patients.

## 7 Conclusions

### 7.1 Summary

This review delves into the intricate bidirectional relationship between narcotic drugs and metabolic syndrome, elucidating the primary effects and underlying mechanisms of both. Narcotic drugs exert multifaceted influences, impacting insulin signalling pathways, lipid metabolism, blood pressure regulation and inflammatory responses. By modulating key molecules in the insulin signalling pathway, altering lipid metabolism, regulating blood pressure and modulating inflammatory responses, narcotic drugs can significantly affect the pathophysiology of metabolic syndrome. Conversely, the influence of metabolic syndrome on the efficacy and safety of anaesthetic drugs is substantial, with the patient's metabolic state profoundly altering drug metabolism and response.

Key areas of investigation include the PI3K/Akt pathway in insulin signalling, lipid metabolism regulation, blood pressure control mechanisms and systemic inflammatory responses. Through these mechanisms, narcotic drugs influence the overall health of patients with metabolic syndrome, while the presence of metabolic syndrome can alter the metabolism and effects of anaesthetic drugs. The complexity of this bidirectional relationship underscores the importance of considering the metabolic status of patients in the management of anaesthesia to achieve optimal therapeutic outcomes and ensure safety.

### 7.2 Outlook

Future research must advance understanding of the bidirectional interplay between anaesthetics and metabolic syndrome (MS), translating mechanistic insights into precision care. Key priorities include developing subtype‐specific anaesthetic strategies (e.g., for insulin resistance‐ or hypertension‐dominant MS) paired with biomarker‐guided dosing models to individualise anaesthetic selection/dosage, optimising efficacy and minimising side effects. Designing MS‐compatible anaesthetics targeting metabolic pathways to reduce anaesthesia‐induced metabolic disturbances is also critical. Validating these requires large‐scale, subtype‐specific trials comparing candidate agents with standards (e.g., propofol, sevoflurane) by assessing long‐term metabolic outcomes and perioperative complications. Rigorous validation of predictive tools will underpin personalised care. Ultimately, deeper insight into anaesthesia‐MS crosstalk will drive precision anaesthesiology, improving perioperative safety and long‐term outcomes for MS patients while advancing both fields.

## Author Contributions

**Yuying Huang:** conceptualization (equal), writing – original draft (equal). **Qinghai Lan:** conceptualization (equal), writing – original draft (equal). **Yijian Chen:** conceptualization (equal), writing – original draft (equal). **Youchun Li:** conceptualization (equal), visualization (equal), writing – original draft (equal). **Baolin Zhong:** conceptualization (equal), visualization (equal), writing – original draft (equal). **Yuxin Zhan:** conceptualization (equal), investigation (equal), writing – review and editing (equal). **Simin Deng:** conceptualization (equal), investigation (equal), writing – review and editing (equal).

## Consent

The authors have nothing to report.

## Conflicts of Interest

The authors declare no conflicts of interest.

## Data Availability

Data sharing is not applicable to this article as no datasets were generated or analysed during the current study.
